# The NUTRIC Score as a Tool to Predict Mortality and Increased Resource Utilization in Intensive Care Patients with Sepsis

**DOI:** 10.3390/nu15071648

**Published:** 2023-03-28

**Authors:** Marek Wełna, Barbara Adamik, Andrzej Kübler, Waldemar Goździk

**Affiliations:** 1 Clinical Department of Anaesthesiology and Intensive Therapy, Wroclaw Medical University, 50-556 Wroclaw, Poland; 2 Department of Anesthesiology and Intensive Therapy, Wroclaw University Hospital, 50-556 Wroclaw, Poland

**Keywords:** intensive care, sepsis, nutrition, mortality prediction

## Abstract

The Nutrition Risk in Critically Ill score (NUTRIC) is an important nutritional risk assessment instrument for patients in the intensive care unit (ICU). The purpose of this study was to evaluate the power of the score to predict mortality in patients treated for sepsis and to forecast increased resource utilization and nursing workload in the ICU. The NUTRIC score predicted mortality (AUC 0.833, *p* < 0.001) with the optimal cut-off value of 6 points. Among patients with a score ≥ 6 on ICU admission, the 28-day mortality was 61%, and 10% with a score < 6 (*p* < 0.001). In addition, a NUTRIC score of ≥6 was associated with a more intense use of ICU resources, as evidenced by a higher proportion of patients requiring vasopressor infusion (98 vs. 82%), mechanical ventilation (99 vs. 87%), renal replacement therapy (54 vs. 26%), steroids (68 vs. 31%), and blood products (60 vs. 43%); the nursing workload was also significantly higher in this group. In conclusion, the NUTRIC score obtained at admission to the ICU provided a good discriminative value for mortality and makes it possible to identify patients who will ultimately require intense use of ICU resources and an associated increase in the nursing workload during treatment.

## 1. Introduction

The NUTRIC score (nutrition risk in the critically ill) is a risk assessment tool. It was developed for use in patients treated in the intensive care unit (ICU) in order to help identify those who would benefit most from nutritional therapy [1]. The benefit of using the tool is estimated by calculating the change in the survival rate; a lower NUTRIC score is associated with lower mortality, which has been confirmed in various groups including critically ill COVID-19 patients [2,3], mechanically ventilated ICU patients [4,5] patients after cardiothoracic surgery treated in a recovery unit [6], and in patients with severe community-acquired pneumonia [7]. The conceptual model used to develop the NUTRIC score was complex and consisted of parameters describing chronic and acute inflammation, age, general clinical assessment, organ failure assessment, and markers of acute and chronic starvation. The model was externally validated and a good discrimination confirmed its predictive ability [8]. Several further studies have shown an association between the NUTRIC score and ICU mortality and it has been observed that, especially patients with high NUTRIC scores may benefit from optimal nutrition, thus improving survival. [9,10,11].

Validation of the NUTRIC score can be seen primarily in the impact on 28-day mortality or the requirement for prolonged mechanical ventilation in ICU patients [1,12]. However, the relationship between the score and the need to use other ICU resources has not been assessed. A group of patients who require a much greater use of resources during an ICU/hospital stay are patients with sepsis [13,14]. The analysis done by Jones et al. showed that patients hospitalized for sepsis consumed significantly more hospital resources over a 12-month period compared to patients hospitalized for diseases other than sepsis [15]. Full access to hospital resources is extremely important in the context of a rapid, unexpected overburden of the ICU, which was experienced during the COVID-19 pandemic. However, a steady increase in ICU resource requirements had been documented even before the global COVID-19 pandemic [16]. The main drivers of the growing demand for intensive care have been observed to be the ageing of the population, the increasing number and complexity of surgical interventions, the implementation of new therapies, and increasing public expectations for the availability and effectiveness of healthcare based on improved outcomes. Therefore, an additional predictive tool may be useful to make better use of resources in intensive care units. The relationship between the NUTRIC score and the need to use more ICU resources in hospitalized sepsis patients has not been assessed.

The aim of this study was to assess the power of the NUTRIC score in predicting 28-day mortality in patients with sepsis, since a worse prognosis on admission may be associated with the need for more intense use of ICU resources and a correspondingly higher nursing workload. We hypothesized that NUTRIC scoring could have additional use as a tool for predicting increased resource utilization in the ICU.

## 2. Materials and Methods

### 2.1. Patient Population

This study was a secondary analysis of prospectively collected data from a sepsis registry database. The database was built using the clinical and demographic data of patients treated in a 25-bed mixed ICU that provides care in a 996-bed university hospital. The study included adults (>18 years of age) diagnosed with sepsis or septic shock, admitted from January to December 2014. The exclusion criteria were re-admission to the ICU or stay in the ICU for less than 24 h. During the study period, a total of 332 consecutive patients admitted to the ICU were screened for inclusion/exclusion criteria. A total of 156 patients met the inclusion criteria. There were 10 cases of incomplete data, so 146 patients were included in the final analysis.

### 2.2. Ethics

The study was approved by the local ethics committee at the Wroclaw Medical University, approval number KB 23/2015, and complies with the Declaration of Helsinki of the World Medical Association. The need for informed consent was waived due to the retrospective, observational nature of the study.

### 2.3. Patient Management and Data Collection

All patients in the study received standard treatment for sepsis or septic shock according to the Surviving Sepsis Campaign guidelines that were in use at the time in the ICU [17]. At least three specialists in anesthesiology and intensive care and four residents provided care for ICU patients during day shifts from 7 a.m. to 3 p.m. Later, care was provided by one specialist in anesthesiology and intensive care and two residents. The nurse-to-patient ratio of was 1:1 or 1:2 depending on the stability of the patient. For the purpose of this study, the SEPSIS-III criteria were applied retrospectively to the sepsis registry database [18].

Due to the unavailability of the IL-6 records in the database, the previously validated, modified version of the scoring (mNUTRIC score) was used for the calculations on the tested sample [1]. The following data collected in the sepsis registry database were used for the calculation of the mNUTRIC score in the present study: age, baseline Acute Physiology and Chronic Health Evaluation II (APACHE II) score, baseline Sequential Organ Failure Assessment (SOFA) score, number of comorbidities, and days from hospital admission to ICU admission. The mNUTRIC score was calculated on admission to the ICU. The APACHE II and SOFA scores were calculated in accordance with source publications [19,20]. Both scores are routinely used for evaluating the severity of the clinical status of patients at the ICU. The detailed scoring system used to calculate mNUTRIC score is presented in Table 1.

In addition, the treatment requirements during the ICU stay were registered in the database and the resource-consuming interventions were the focus of this analysis. The following procedures for managing each patient during the ICU stay were noted: resuscitation and vasoactive medications; mechanical ventilation; renal replacement therapy; nutrition therapy; the administration of corticosteroids, anticoagulants, and blood products; glucose control; and surgery. The quantification of the daily nursing workload for each patient was calculated using the Therapeutic Intervention Scoring System-28 (TISS-28) and the mean index value was recorded in the database. In our ICU, the TISS-28 score is routinely used as a nursing workload and severity measurement system [21].

### 2.4. Statistical Analysis

Descriptive statistics included median (interquartile range) for quantitative variables, and frequency (percentage) for qualitative variables. There were no missing data in the database. The T-student test was used for the comparison of continuous variables between the study groups. Categorical variables were analyzed with the chi-squared test, and contingency tables were used to analyze the frequency distribution of the categorical variables. Receiver operating characteristic (ROC) curve analysis was used to measure the ability of the NUTRIC score to discriminate between death and survivors by calculating the area under the curve (AUC), including 95% confidence intervals (CI), to determine the sensitivity and specificity; the results of the Younden statistics indicated an optimal cut-off value for the NUTRIC score. The Kaplan–Meier curves and the log-rank test were used to assess differences in 28-day survival functions based on the value of the NUTRIC score. Univariate logistic regression was performed to compare the predictive power of clinical scales (NUTRIC, APACHE II alone and SOFA score alone) for prognosing 28-day mortality; the results were reported as the odds ratio (OR) with 95% confidence intervals (CI). Multivariate logistic regression analysis was performed to create a model predicting 28-day mortality. The association between NUTRIC score and selected covariates (gender, concomitant illnesses, lactate level, procalcitonin level, hypoglycemia, need for renal replacement therapy, the need for renal replacement therapy, the need for respiratory support, positive blood culture, time to antibiotic administration) and mortality was assessed; the results were reported as odds ratios (OR) with 95% confidence intervals (CI). All statistical analysis was carried out with Statistica, version 13 (StatSoft Inc., Tulsa, OK, USA). A *p* value < 0.05 was considered statistically significant.

## 3. Results

### 3.1. Study Sample

During the study period, a total of 332 consecutive patients admitted to the ICU were screened for inclusion/exclusion criteria. Of this, 156 patients met the inclusion criteria. Due to incomplete data, 146 patients were included in the final analysis. The median age in the sample was 66 (IQR 58–77) and the majority of patients were male (n = 95, 65%). Out of 146 septic patients enrolled, 129 (88%) had septic shock on admission to the ICU. The main causes of sepsis and septic shock were pneumonia (49%), intra-abdominal infection (35%), and urinary tract infection (7%). Patients were transferred from general wards (48%), an operating theater (38%), the emergency department (7%), other hospitals (6%), and the high dependency unit (1%). Most of the patients were admitted in critical condition with the median SOFA score calculated on day 1 at the level of 10 points (IQR 7–13) and with an APACHEII score of 21 points (IQR 15–27). The severity of the clinical condition of the patients was also reflected in the high proportion of cases with failure of four or more organs diagnosed on admission to the ICU (51%). The median ICU stay was 10 days (IQR 4–23), and the hospital stay was 32 days (IQR 11–55). Baseline patient characteristics are presented in Table 2.

### 3.2. The Optimal Cut-Off Point for mNUTRIC

**Figure 1:**
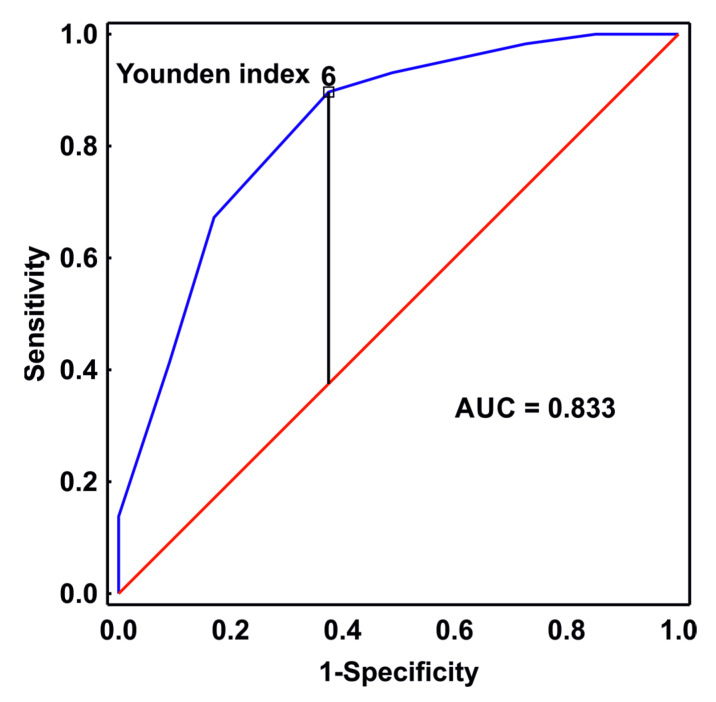
The receiver operating characteristic (ROC) curve illustrates the diagnostic ability of the mNUTRIC score calculated in patients with sepsis on admission to the ICU as a predictor of 28-day survival with an area under the curve (AUC) of 0.833 (95% CI 0.76–0.89, *p* < 0.001). The results of analyzing the Younden statistics indicated the optimal cut-off value for the mNUTRIC score at 6 points.

First, we assessed the prognostic performance of the mNUTRIC-score for 28-day mortality. The mNUTRIC score is a 9-point scale. In the ROC curve analysis, the mNUTRIC score calculated on admission to the ICU had the ability to predict 28-day mortality with an AUC of 0.833 (95% CI 0.76–0.89, *p* < 0.001). This is shown in Figure 1. The optimal cut-off value for the mNUTRIC score was 6 points, with a sensitivity of 90% and specificity of 63%, and this point was used to divide the study sample for further analysis, i.e., the group of patients with an mNUTRIC score ≥ 6 points and the group with a mNUTRIC score < 6 points. Among patients with mNUTRIC score ≥ 6 on ICU admission, 28-day mortality was 61%, and with score < 6, mortality was 10% (*p* < 0.001).

### 3.3. mNUTRIC Predictive Power

Then, a one-way logistic regression analysis was performed to compare the predictive power of the clinical scales computed on admission to the ICU. The mNUTRIC predicted 28-day mortality with an odds ratio of 2.24 (95% CI 1.71–2.95, *p* < 0.001), APACHE II alone with an OR 1.21 (95% CI 1.13–1.29, *p* < 0.001), and SOFA score alone with an OR 1.43 (95% CI 1.26–1.63, *p* < 0.001)

### 3.4. Characterization of Groups of Patients According to the Optimal Cut-Off Point for the mNUTRIC Score

Based on the result of the ROC curve analysis and the results of the Younden statistics, the study sample was divided into Group 1 (patients with an mNUTRIC score < 6 points, n = 61) and Group 2 (patients with an mNUTRIC score ≥ 6 points, n = 85). In the mNUTRITIC < 6 group, 64% were male, and in the mNUTRITIC ≥ 6 group it was 66% (*p* = 0.807). The analysis of the variables used to calculate the mNUTRIC score is shown in Table 3.

### 3.5. Patient Management

The nursing manpower in the care of patients was evaluated with the TISSS-28 score index, and the obtained results indicated that the nursing workload was significantly greater in the group with an mNUTRIC score ≥ 6 points, compared with an mNUTRIC score of <6 points (TISS-28: 36 points, IQR 33–40 vs. 31 points, IQR 28–34, *p* < 0.001). In patients with an mNUTRIC score ≥ 6 points, septic shock was diagnosed in 96% of cases and with an mNUTRIC < 6 points in 78% (*p* = 0.001). Fluid resuscitation was used in the majority of patients in both groups (*p* = 0.154) and vasopressors had to be administered in almost all patients with an mNUTRIC ≥ 6 points (98%) and in 82% of cases with an mNUTRIC score < 6 points (*p* = 0.001). Mechanical ventilation and renal replacement therapy were more often employed in the treatment of patients with an mNUTRIC ≥ 6 points than in cases with an mNUTRIC score < 6 points (99 vs. 82% and 54 vs. 26%, respectively). Therapy with steroids was required more than twice as often in the group with an mNUTRIC ≥ 6 points, compared with an mNUTRIC < 6 points (68 vs. 31%), and the need for blood products was also much higher (60 vs. 43%). Table 4 compares the frequencies of different treatment requirements on the day of admission to the ICU.

### 3.6. Patient Management

**Figure 2:**
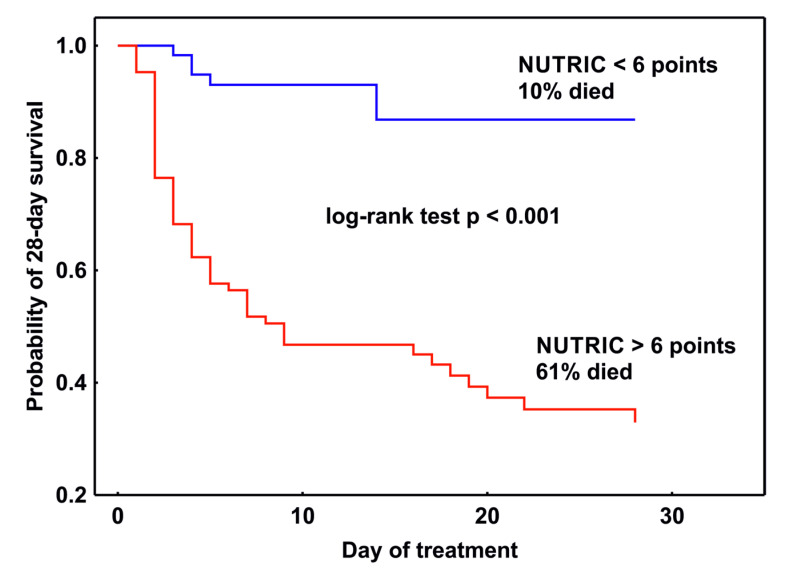
Comparison of 28-day survival in 2 groups of patients with sepsis on admission to the ICU based on the calculated value of the mNUTRIC score.

The 28-day mortality of the entire study group was 40%. Among the patients who died, the NUTRIC score calculated on ICU admission was ≥6 points in 90% of cases. The median value of the mNUTRIC score in Survivors was 4 points (IQR 3–6), and in non-survivors 7 (IQR 6–8). The Kaplan–Meier 28-day survival analysis of time to death showed that there was statistical significance between groups with an mNUTRIC score <6 and ≥ 6 points (*p* < 0.001, log-rank test), (Figure 2).

In addition, a multivariate logistic regression analysis was performed to create a model to predict 28-day mortality. The model’s backward selection determined the choice of the variables from the set of parameters assessed on ICU admission (mNUTRIC score, gender, lactate level, procalcitonin level, need for renal replacement therapy, need for respiratory support, positive blood culture, time to antibiotic administration, and co-morbidities). The baseline mNUTRIC score (OR = 1.86; 95%CI 1.36–2.54), presence of septic shock on admission to the ICU (OR = 4.19, 95%CI 1.38–12.73), and lactate level (OR = 1.32; 95%CI 1.08–1.59) were significant predictors of 28-day mortality; other parameters did not enter the model. The results are presented in Table 5.

## 4. Discussion

The results of our study showed that in the population of patients with sepsis, those who had an mNUTRIC score ≥ 6 on ICU admission were identified as having a significantly lower chance of survival. Moreover, an mNUTRIC score of ≥6 was associated with a more intense use of ICU resources, as evidenced by a significantly higher proportion of cases requiring mechanical ventilation, renal replacement therapy, and vasopressor support. Steroid therapy was also required more than twice as often in the group with an mNUTRIC ≥ 6 points, and the demand for blood products was also significantly higher. The nursing workload quantified on the TISS-28 scale was significantly greater in the group with an mNUTRIC score ≥ 6 points compared to the care requirement in patients with an mNUTRIC < 6 points. Thus, already on admission to the ICU, it could be assumed that the treatment of a patient with sepsis with an mNUTRIC score ≥ 6 points would require more intensive use of ICU resources. To our knowledge, this is the first study to assess the utilization of therapeutic resources in the treatment of sepsis patients based on the mNUTRIC score.

The NUTRIC score is a novel risk assessment tool initially developed to help identify patients who are more likely to benefit from nutritional therapeutic interventions in the ICU setting [1]. The conceptual model for the development of the NUTRIC score incorporated predictor markers of acute starvation, chronic starvation, acute inflammation, and chronic inflammation [22]. In contrast to many widely used nutritional risk assessments, this score was developed specifically for use in patients admitted to the intensive care unit [23,24,25,26,27,28,29,30]. One potential drawback of the mNUTRIC score originates from the characteristics of the study group that were used to develop the score; the study was based on a relatively small group (n = 597) consisting mainly of Caucasian patients (90%) [1]. These potential disadvantages were eliminated in a series of studies conducted in various geographical regions, involving larger cohorts of patients of different race and social status [31]. The mNUTRIC score accurately predicted 28-day mortality in a randomized control trial of 1199 mechanically ventilated ICU patients completed in Canada [8]. A similar group of critically ill patients requiring mechanical ventilation was included in a study conducted in Argentina, and the obtained results confirmed the relationship between higher mortality and increased mNUTRIC score [32]. Another study in Portugal, which analyzed the outcomes of 1143 adult ICU patients, also supported the above-mentioned findings: a high mNUTRIC score was associated with longer hospitalization, fewer days free of mechanical ventilation, and higher 28-day mortality [33]. Similar conclusions were drawn in the Indian study, where significant differences were noted between the high and low NUTRIC groups in terms of mortality (*p* < 0.001), ICU length of stay (*p* < 0.014), and duration of mechanical ventilation (*p* < 0.001) [4]. Data from the Singapore study confirmed the association of a high mNUTRIC score with hospital mortality [34]. These findings strongly suggest that the mNURTRIC score, when used as a predictor of mortality, performs well across cohorts studied in different geographic regions, with different race and social status. Our research also confirmed earlier observations.

A major practical drawback of the original NUTRIC score was the inclusion of the concentration of interleukin 6 in the scoring, as this parameter is not routinely measured in ICU patients and is often unavailable in hospital databases. Therefore, a modified version of the score (mNUTRIC) was later proposed, without taking into account the value of the interleukin 6 concentration [1]. The mNUTRIC score was externally validated using data from a randomized clinical trial database of 1223 mechanically ventilated ICU patients [35]. The external validation was repeated in a later study by Rahman et al., who found that the probability of death at day 28 increased by 1.4 (95% CI, 1.3–1.5) for every point increase on the mNUTRIC score, confirming the relationship between the mNUTRIC score and mortality [8]. In the present study the modified version of the NUTRIC score was used. In the analyzed cohort, a very good performance of the mNUTRIC score was found in predicting 28-day mortality with an AUC of 0.833 (95% CI 0.76–0.89, *p* < 0.001), and the optimal cut-off value of 6 points was identified with the Younden statistics and used for dividing the studied sample for further analysis. The group with an mNUTRIC score ≥ 6 points had a significantly higher mortality compared to the group with <6 points (81% versus 28%, *p* < 0.001). Previously, de Vries et al. validated the predictive ability of the mNUTRIC score using a cohort of 475 mechanically ventilated patients admitted to an ICU in the Netherlands between 2011 and 2013, and a good discrimination capacity of the tested score was confirmed in the ROC analysis with an AUC of 0.768 (95% CI 0.722–0.814) [12]. Similar results were obtained by Mukhopadhyay et al. in an Asian population of ICU patients, with an AUC of 0.71 for predicting mortality using the mNUTRIC scale [10]. These results confirmed the ability of the mNUTRIC score to predict mortality in a specific subset of patients with sepsis and septic shock, i.e., a group of patients at high risk of death.

A high prevalence of malnutrition (38% to 78%) has been reported in patients in intensive care units (ICU), which was associated with increased morbidity and poor outcome [36]. Malnutrition may have a negative impact on the immune response during sepsis, increasing ICU mortality and length of hospital stays [37,38]. Numerous studies have confirmed the usefulness of the mNUTRIC score in identifying patients from the high nutritional risk group in the population of ICU patients [1,8,10,39]. This subpopulation would benefit most from appropriate nutritional therapy. According to a study by Hung et al., immunocompetent patients were more likely to be affected by inadequate nutrition, and patients with unmet caloric needs had the worst prognosis, with a 90-day mortality rate of more than 90% [40]. In contrast, in immunocompromised sepsis patients, insufficient nutrition had no effect on mortality. The mNUTRIC score did not distinguish between immunocompromised and immunocompetent patients, but the surviving patients had lower SOFA and NUTRIC scores and higher caloric meets than non-survivors. There is a shortage of large studies evaluating the outcome of septic patients subjected to different caloric regimes. Retrospective analysis of caloric and protein consumption in 1171 critically ill patients (22.8% sepsis) confirmed that both under and overnutrition were harmful [41]. This finding was not confirmed in the TARGET trial, which evaluated the effect of delivering 1.5 kcl/mL vs. 1 kcl/mL of enteral nutrition per kg of body weight in mechanically ventilated ICU patients. Sepsis was present in 25% of each treatment group. Patients in energy dense group received significantly higher calorie delivery. It did not affect mortality, liberation of organ support, or incidence of infective complications [42].

A recently published study found that 48.9 million cases of sepsis were reported worldwide in 2017; there were also 11 million deaths related to sepsis in the same year [43]. These estimates are based on a unique, detailed analysis of death certificates and are global estimates of the incidence of sepsis, including cases of sepsis that have not been treated in a hospital. The highest incidence and mortality from sepsis were estimated in regions with the lowest availability of medical resources, indicating the need for administrative tools to improve ICU resource utilization. So far, a lot of research has been done to find a model to improve access to ICU resources. One of the available methods is the prediction of the ICU length of stay, assuming that this parameter can be related to the intensity of ICU resource utilization. Verburg et al. provided a systemic review of models designed to predict the ICU length of stay, and 11 different models were identified and investigated [44]. The most frequently used predictors in these models were overall disease severity, source of admission, age, use of mechanical ventilation, the Glasgow Coma score, comorbidities, and organizational predictors. Unfortunately, no model has been assessed as fully competent for planning and identifying unexpectedly long ICU stay or for benchmarking purposes. Another approach to improving access to ICU resources is to use disease-specific severity scores. The PIRO (predisposition, insult, response, organ dysfunction) score was used to assess severity and predict resource utilization in ventilator-associated pneumonia (VAP) [45]. The model was designed as a simple, practical clinical tool for predicting health-care resource utilization based on the length of ICU stay and the duration of mechanical ventilation, and demonstrated greater use of medical resources in patients with high and very high risk of death based on the PIRO prognosis. Yet another approach to predicting the use of ICU resources may be a model designed to predict the use of specific invasive therapies. Recently, Sukmark et al. developed a simplified scoring system for predicting major adverse kidney events among patients diagnosed with acute kidney injury (AKI) and treated in the ICU [46]. This simplified clinical score was based on easily available parameters such as the Glasgow Coma scale, tachypnea, vasopressor use, mechanical ventilation use, oliguria, serum creatinine, blood urea nitrogen, hematocrit, and thrombocytopenia. The model performance was adequate when internally validated (AUC under the ROC curve of 0.80) and feasible even in resource-limited settings; however, the model has not yet been externally validated. Various models for predicting shock and vasopressor use have also been developed and evaluated. Recently, Kwak et. al. used an attention-based deep learning model to predict the need for vasopressor therapy during the first 24 h of ICU stay. Only vital signs were used in the final model, with heart rate, respiratory rate, and mean arterial pressure contributing the most (AUC of 0.83) [47]. In a study by Liu et. al., a clustering technique, called fuzzy c-means, was employed to develop a model predicting vasopressor requirements for critically ill patients (AUC of 0.81) [48]. The authors have suggested the existence of a pre-shock state preceding the transition from sepsis to septic shock; detecting this state with the help of the developed model may be useful in resource allocation, especially when ICU availability is constricted. Later, the Medical Information Mart for Intensive Care–III database was used to externally validate the model. Three different machine learning techniques were used, yielding good performance in identifying septic patients who could develop septic shock, with an AUC of 0.93 and median early warning time of 7 h [49,50]. A similar approach was employed for predicting the need for intubation [51,52,53]. Siu et al. used machine learning to develop a model predicting the need for intubation during the first 24 h after ICU admission. The parameters required for the model were as follows: blood gas results, the Glasgow Coma Score, respiratory rate, oxygen saturation, temperature, age, and parameters of oxygen therapy. The reported AUC of the model was 0.86 (95% CI 0.85–0.87) [50].

The approaches presented above often required advanced automated electronical real-time data collection and analysis. This kind of know-how is not always available in an ICU setting. We employed the mNUTRIC score as a simple tool for predicting ICU resource utilization. It is noteworthy that all the parameters necessary to calculate the mNUTRIC (age, baseline APACHE II score, baseline SOFA score, number of comorbidities, and days from hospital admission to ICU admission) are routinely collected or calculated and stored in the hospital records of patients. Using the cut-off point of 6 on the 9-point mNUTRIC scale, we were able to identify a group of patients who significantly more often required intensive use of ICU resources during their entire stay in the ICU.

## 5. Limitations

In numerous previous studies it has been observed that the mNUTRIC score identifies patients at high risk of malnutrition who are likely to benefit from nutritional therapy during their ICU stay [1,8,10,39]. Due to the lack of specific nutritional data, the relationship between mortality, nutritional adequacy, and the mNUTRIC score was not assessed in the studied cohort and we acknowledge that this as a limitation of the study. With the development of intensive care, the complexity of the organization and structure of these departments has also increased; therefore, the development and use of scoring systems can contribute to improving the allocation of material and human resources. Nursing workload quantification indices are nowadays one of the fundamental tools in ICU planning and evaluation. Our results indicate the usefulness of the mNUTRIC score as a potential tool for predicting increased resource utilization in the ICU; however, it is a single center analysis with a relatively small sample size, and we consider this to be another limitation of the study.

## 6. Conclusions

The mNUTRIC score obtained at admission to the ICU provided a good discriminative value for 28-mortality and makes it possible to identify patients who will ultimately require intense use of ICU resources with an associated increase in the nursing workload during ICU sepsis treatment. Our data indicate that the mNUTRIC score may be useful in ICU resource planning, especially in the face of increased demand for intensive care services such as during a global pandemic. However, external validation based on a larger cohort is required before advocating for the wider use of the mNUTRIC score as an additional tool for ICU resource planning.

## Figures and Tables

**Table 1:** Variables used to calculate the mNUTRIC score. The score on the mNUTRIC scale ranges from 0 to 10 points.

Variable	Range	Points
Age (years)	Below 50	0
	From 50 to 74	1
	75 and more	2
APACHE II (points)	Below 15	0
	From 15 to 19	1
	From 20 to 27	2
	28 and more	3
SOFA (points)	Below 6	0
	From 6 to 9	1
	10 and more	2
Co-morbidities (n)	0, 1	0
	2 and more	1
Days before ICU admission	0	0
	1 and more	1

**Table 2:** Baseline patient characteristics according to the NUTRIC score.

	mNUTRIC ≥ 6 pts	mNUTRIC < 6 pts	*p*-Value
	(n = 85)	(n = 61)	
Age (years)	69 (63–80)	61 (50–78)	<0.001
Male/Female	56 (/29)	(39/22)	0.807
APACHE II	24 (21–30)	14 (11–18)	<0.001
SOFA	12 (10–15)	8 (6–10)	<0.001
Source of infection (%)			
Lungs	49	48	0.823
Abdominal cavity	31	30	0.888
Urinary tract	4	11	0.062
Other *	16	11	0.396
Co-morbidities (%)			
Chronic circulatory failure	39	15	0.001
Liver disease	11	3	0.088
Hematological diseases	5	6	0.446
Hypertention	52	36	0.060
Diabetes	25	21	0.632
Copd	9	2	0.051
Chronic kindey disease	22	11	0.090
Malignancies	13	10	0.564
Procalcitonin (ng/mL)	6.2 (2.75–28.6)	4.54 (0.89–8.75)	0.174
Lactate (mmol/L)	3.96 (1.87–7.88)	1.69 (1.16–3.2)	<0.001
LOS before ICU (day)	3 (1–8)	2 (0–13)	0.112
LOS in the ICU (day)	8 (3–18)	13 (7–29)	0.003
LOS in the hospital (day)	16 (6–43)	43 (28–65)	<0.001
28-day mortality (%)	61	10	<0.001

* including wound infection, skin infection, central nervous system infection, bloodstream infection, bones and joints infection, unknown. COPD, chronic obstructive pulmonary disease.

**Table 3:** Analysis of the variables used to calculate the mNUTRIC score.

	mNUTRIC ≥ 6 pts	mNUTRIC < 6 pts	*p*-Value
	(n = 85)	(n = 61)	
Age (years)	69 (63–80)	61 (50–78)	<0.001
APACHE II	24 (21–30)	14 (11–18)	<0.001
1st day SOFA	12 (10–15)	8 (6–10)	<0.001
LOS before ICU admission	3 (1–8)	2 (0–13)	0.112
Number of comorbidities	2 (1–3)	1 (1–2)	<0.001

APACHE II, Acute Physiology and Chronic Health Evaluation II; SOFA, Sequential Organ Failure Assessment; ICU, intensive care unit; LOS, length of stay. Data are presented as median (interquartile range). The *p*-value represents differences between the groups.

**Table 4:** Comparison of therapeutic resource requirements for treating septic patients with an mNUTRIC score < 6 and ≥ 6 points.

Parameter	NUTRIC ≥ 6	NUTRIC < 6	*p*-Value
Fluid resuscitation n (%)	73 (86)	46 (75)	0.154
Vasopressors n (%)	83 (98)	50 (82)	0.001
Mechanical Ventilation n (%)	84 (99)	53 (87)	0.018
RRT n (%)	46 (54)	16 (26)	0.001
Steroids n (%)	58 (68)	19 (31)	0.001
Nutrition Theraphy n (%)	48 (56)	45 (74)	0.021
Enteral n (%)	31 (36)	28 (46)	0.252
Parenteral n (%)	17 (19)	17 (26)	0.286
Insulin n (%)	50 (59)	38 (62)	0.643
Thromboprohylaxis n (%)	67 (79)	58 (95)	0.001
Blood products n (%)	51 (60)	26 (43)	0.038
Surgery during ICU stay n (%)	29 (34)	20 (33)	0.866

RRT, renal replacement therapy; ICU, intensive care unit. Data are presented as frequency (percentage). The *p*-value represents differences between the groups.

**Table 5:** Univariate and multivariate logistic regression analysis of the predictors of 28-day mortality. All parameters refer to the day of ICU admission.

	Univariate Analysis			Multivariate Analysis		
Parameter	Odds Ratio	95%CI	*p*-Value	Odds Ratio	95%CI	*p*-Value
mNUTRIC score	2.24	1.71–2.95	<0.001	1.86	1.36–2.54	<0.001
Septic shock	8.24	3.59–8.89	<0.001	4.19	1.38–12.73	0.011
Lactate level	1.52	1.27–1.82	<0.001	1.32	1.08 –1.59	0.005
Gender	0.65	0.32–1.33	0.248			
Procalcitonin level	1.00	0.99–1.01	0.990			
RRT	3.47	1.72–7.01	<0.001			
Respiratory support	1.78	0.32–0.22	0.521			
Positive blood culture	1.02	0.48–2.15	0.949			
Time to antibiotic administration	1.00	0.99–1.00	0.572			
Co-morbidities:						
Chronic circulatory failure	2.07	0.10–4.30	0.048			
Liver disease	1.91	0.55–6.59	0.302			
Hematological diseases	0.91	0.20–3.94	0.894			
Hypertention	1.37	0.71–2.68	0.345			
Diabetes	1.08	0.49–2.36	0.843			
Copd	2.67	0.61–11.64	0.191			
Chronic kindey disease	2.01	0.85–4.74	0.108			
Malignancies	1.60	0.56–4.53	0.376			

RRT, renal replacement therapy; COPD, chronic obstructive pulmonary disease. CI, confidence intervals.

## Data Availability

Restrictions apply to the availability of these data. Data were obtained from a local sepsis registry and are available from the corresponding author with the permission of sepsis registry group.
